# Magnetic Resonance Imaging Studies on Acupuncture Therapy in Depression: A Systematic Review

**DOI:** 10.3389/fpsyt.2021.670739

**Published:** 2021-08-20

**Authors:** Jinhuan Zhang, Xiaoxiong Wu, Dehui Nie, Yuanyuan Zhuo, Jiaying Li, Qingmao Hu, Jinping Xu, Haibo Yu

**Affiliations:** ^1^The Fourth Clinical Medical College of Guangzhou University of Chinese Medicine, Shenzhen, China; ^2^Institute of Biomedical and Health Engineering, Shenzhen Institutes of Advanced Technology, Chinese Academy of Sciences, Shenzhen, China; ^3^Acupuncture Department, Shenzhen Traditional Chinese Medicine Hospital, Shenzhen, China; ^4^CAS Key Laboratory of Human-Machine Intelligence-Synergy Systems, Shenzhen Institutes of Advanced Technology, Chinese Academy of Sciences, Shenzhen, China; ^5^School of Artificial Intelligence, University of Chinese Academy of Sciences, Beijing, China

**Keywords:** magnetic resonance imaging, acupuncture, depression, treatment, systematic review

## Abstract

Accumulating studies had been performed using magnetic resonance imaging (MRI) to understand the neural mechanism of acupuncture therapy for depression. However, inconsistencies remain due to differences in research designs and MRI analytical methods. Therefore, we aim to summarize the current MRI research and provide useful information for further research by identifying papers published in English and Chinese about MRI studies on acupuncture for depression up to November 2020. A total of 22 studies met the inclusion criteria, including 810 depression patients and 416 health controls (HCs). The applied designs of these studies are mainly random control trial and pre–post designs. The MRI analytical methods are mainly (fractional) amplitude of low-frequency fluctuation (fALFF/ALFF) and functional connectivity (FC), whereas a small subset of studies used voxel-based morphometry (VBM) and diffusion tensor imaging (DTI). The most consistent functional MRI (fMRI) results showed increased *N*-acetylaspartate/creatine (NAA/Cr) ratios, increased ALFF in the right precuneus, decreased ALFF in the inferior frontal gyrus (IFG), and increased FC of the anterior cingulate cortex (ACC). In contrast, no significant neurological changes were identified in any of the DTI or VBM studies. However, clear, reliable conclusions cannot be drawn due to the use of different designs, analytical methods, seed points selected, types of depression, acupuncture points, and so on. Improved report specifications, well-designed studies, consistent analytical methods, and larger sample sizes will enable the field to better elucidate the underlying mechanisms of acupuncture in depressed patients.

## Introduction

Depression is a common mental illness, which has been recognized as a major public health problem that has a substantial impact on an individual's ability to function within daily and societal environments (1). Depressed patients may lose interest in physical activity; lose their appetite or overeat; have difficulty concentrating, remembering details, or making decisions; and more seriously may attempt suicide or commit suicide (2). In various studies, the rate of depression or depressive symptoms among students varied from 1.4 to 73.5% (3, 4), and those with suicidal ideation varied from 4.9 to 35.6% (5, 6). Major depressive disorder (MDD) is the most common and severe mental disorder with a lifetime prevalence of 6–15% (7, 8).

Antidepressant medication may be provided as an initial primary treatment for MDD, but they are far from satisfactory due to undesirable side effects and a delay in the onset of therapeutic action (9–11). Faced with limitations of conventional treatments, patients suffering from depression often seek alternative forms of treatment, such as acupuncture therapy (12), one of the world's oldest recognized medical treatments, which has been used to relieve pain and treat mental illness (13, 14) for 1,000's of years. Indeed, several systematic reviews (1, 15, 16) have shown that a single acupuncture therapy session or combination of acupuncture with a suitable adjunct was significantly effective in reducing the severity of depression. In traditional Chinese medicine (TCM) theory, the pathogenesis of depression is the stagnation of liver qi, and acupuncture can regulate qi and mental state. Experimental studies indicated that most of the action of antidepressant effects of acupuncture is mediated *via* the central nervous system (17). Moreover, an increasing number of animal experiment researches identified that the effective mechanism of acupuncture for depression may be through regulation of the hypothalamic–pituitary–adrenal axis (18, 19), neurotransmitter (20–22), anti-inflammatory (23–25), and signaling pathways (26–29).

However, as we all know, humans are complex animals, and the mechanism of acupuncture in treating depression may be different between animal and human studies. Therefore, it is very necessary to investigate the effects of acupuncture on depression at the human brain level. In recent years, magnetic resonance imaging (MRI), due to its minimal invasiveness, lack of radiation exposure, excellent spatial resolution, and relatively wide availability, has been widely used to quantify how acupuncture affects the function and structure of brain regions as well as brain networks (30). Therefore, it is possible for us to explore changes in brain structure, function, and metabolism about acupuncture for depression. Importantly, with the increase of research in this area, the central mechanism of acupuncture's effect on depression is becoming more and more clear. However, there are few reviews of MRI studies on acupuncture for depression. It is necessary to understand the current state of research for better exploration in the future.

Therefore, with our review, we aim to provide a systematic overview of the existing evidence regarding changes in brain structure, function, and metabolism underlying the effects of acupuncture therapy on depression by summarizing the characteristics, methods, and conclusions of relevant MRI research. A meta-analysis was further performed to identify the most reliable results.

## Materials and Methods

### Search Strategy and Study Selection

We conducted our systematic review in accordance with Preferred Reporting Items for Systematic Reviews and Meta-Analyses (PRISMA) guideline (31). We searched the following four electronic databases for clinical MRI research on acupuncture therapy for depression: PubMed, Wanfang, VIP information database, and China National Knowledge Infrastructure (CNKI) up to October 20, 2020. The keywords were as follows: (1) acupuncture therapy, acupuncture, acupuncture point, body acupuncture, auricular acupuncture, electroacupuncture, moxibustion; (2) depression, depressive disorder, major depression disorder; and (3) MRI, magnetic resonance imaging, resting state, fMRI, rs-fMRI, functional connectivity, task fMRI, BOLD, blood oxygen level-dependent, ReHo, ALFF, fALFF, voxel-based analysis, VBM, voxel-based morphometry, Freesurfer, surface based morphometry, cortical thickness, surface area, cortical volume, gray matter volume, gray matter density, GMV, DTI, diffusion tensor imaging, white matter, fractional anisotropy, mean diffusivity, magnetic resonance spectroscopy.

Studies that met the following criteria were included: (1) prospective observational/randomized study; (2) patients with some specific depressive disorder symptoms such as anhedonia (diminished ability to experience pleasure), diurnal variation (i.e., symptoms of depression are worse during certain periods of waking hours), and intensified guilt about being ill (32) and met established diagnostic criteria of depression, including the *Diagnostic and Statistical Manual of Mental Disorders* (DSM) (32), the International Classification of Diseases (ICD) (33), and the Chinese Classification of Mental Disorders (CCMD) (34); (3) the scores of Hamilton Depression Scale (HAMD-17) ≥17 or HAMD-24 ≥20 or standard score of Self-Rating Depression Scale (SDS) ≥53 or total score of Montgomery–Åsberg Depression Rating Scale (MADRS) ≥14; (4) intervention using acupuncture, electroacupuncture (EA), or laser acupuncture (LA); and (5) outcome indicators of brain response assessed using functional MRI (fMRI) or structural MRI (sMRI), and analytical methods are not restricted. Studies with following traits were excluded: (1) protocol, case reports, or case series; (2) other interventions that do not belong to traditional acupuncture, such as transcutaneous electrical nerve stimulation and transcutaneous vagus nerve stimulation; and (3) comorbid severe mental illness or neurological illness. In addition, studies with relatively consistent study design and analytical methods were included in the meta-analysis.

**Figure 1:**
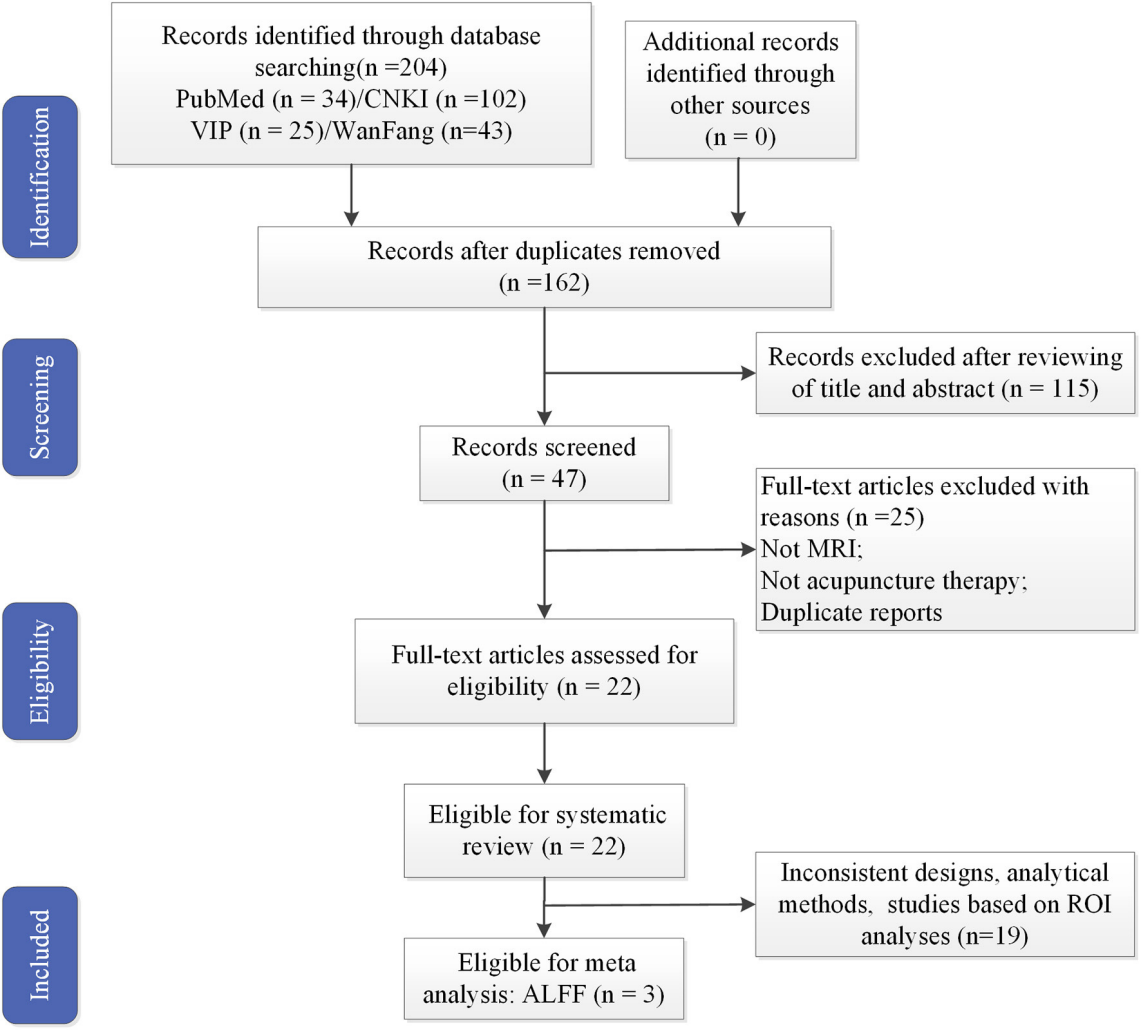
Flowchart of literature selection. MRI, magnetic resonance imaging; ALFF, amplitude of low-frequency fluctuation.

All the identified studies were imported into NoteExpress. After a review of the title and abstracts, studies that did not meet the inclusion criteria were excluded. Final studies were determined after a full-text review. Following this approach, 22 studies were selected (Figure 1).

### Data Extraction

Data were extracted from each study by one reviewer and then verified by a second reviewer. The following key information were extracted from each study: first author, publication year, study design, sample size, characteristics of participants, imaging modality and conditions, analytical methods, main acupoints/sites, and reported results. The peak coordinates and the statistical significance level were extracted from studies with similar MRI analytical methods and designs. Any disagreements in article selection and data extraction were resolved through discussions with a third author.

### Coordinate-Based Meta-Analysis

The present coordinate-based meta-analysis (CBMA) was performed using Signed Differential Mapping with Permutation of Subject Images version 6.21 (SDM-PSI) (https://www.sdmproject.com/). Briefly, CBMA was carried out with the following procedure: collection of peak coordinates and their *t*-values; calculation of the maps of the lower and upper bounds of possible effect sizes; estimation of the map of most likely effect size and its standard error based on the MetaNSUE algorithms; multiple imputations of the maps of the effect size of individual studies; and using a standard random-effects model and Rubin rules to pool the different meta-analyses resulting from the multiple imputations (35–37), and the specific procedures have been extensively described in the SDM-PSI reference manual (https://www.sdmproject.com/manual/).

SDM-PSI is a new-generation algorithm for CBMA. This method has significant improvements in several aspects, such as using threshold-free cluster enhancement (TFCE) statistics, small bias estimates of the overall size estimates, and multiple imputations of the study image, to avoid bias associated with single imputation (35). The detailed data processing procedures are described in our previous article (38). We reported results using uncorrected *p* < 0.005 thresholds with a cluster extent = 10 voxels, since it was found to be optimally balance sensitivity and specificity (36, 39).

## Results

### Study Characteristics

Characteristics of the included studies are reported in Table 1. A total of 22 articles were included in this review. One study was from Australia (46), and the remaining studies were from China. Six (41, 46, 53, 55, 58, 60) were indexed in the Science Citation Index. Four studies (42, 49, 53, 60) adopted one parallel-group design, three studies (40, 44, 57) involved three parallel-arm group designs, and the remaining studies adopted two parallel-arm group designs.

**Table 1:** Demographic and clinical characteristics of included studies.

**References**	**Groups (*n*)**	**Diagnostic criteria**	**Study design**	**Illness duration**	**Symptom severity (baseline)**	**Symptom severity** **(after treatment)**	**Treatments (*n*)**	**Age (years)**	**Main acupoints**	**Treatment frequency** **(each session duration, total period)**	**Scanning instrument/ experimental design**
Duan (40)	Depression (75)	CCMD-3	Observational studies	10.89 ± 5.2 m; 11.94 ± 5.7 m; 13.16 ± 6.7 m	HAMD-24: 30.40 ± 5.95; 29.72 ± 5.83; 31.56 ± 6.21	HAMD-24: 20.72 ± 6.14; 19.10 ± 7.31; 14.54 ± 6.12	Fluoxetine (25); EA (25); fluoxetine+EA (25)	(39.47 ± 11.20); (40.42 ± 10.71); (38.17 ± 11.31)	GV20 and EX-HN3	EA, 30–40 min, once a day, 5 times/w; Fluoxetine 20 mg/d; for 6 weeks.	GE Sigma 3.0T; RS
Duan et al. (41)	MDD (70)	ICD-10	RCT	7.2 ± 2.4 m	HAMD-17: 25.1 ± 3.7; 23.8 ± 4.0	HAMD-17: 12.7 ± 5.5; 10.1 ± 5.1	Fluoxetine+EA (34); fluoxetine (36)	35 ± 8	GV20 and EX-HN3	EA, 30 min once a day; fluoxetine, 20 mg/d; for 6 weeks.	GE Sigma 3.0 T; RS
Wang (42)	Depression (19); HC (19)	CCMD-3	Pre–post	/	MADRS: 21.37 ± 8.32; SAS: 63.88 ± 8.94	MADRS: 8.35 ± 5.71; SAS: 46.18 ± 11.39	MA (19)	(41.68 ± 12.12); (41.05 ± 12.13)	RN12, RN10, RN6, RN4, KL17, and extra-point	MA, once a day for the first 3 days and subsequently once every 3 days for 4 weeks.	Siemens 1.5 T; RS
Yi et al. (43)	Mild depression (18)	DSM-IV	/	More than 1 m	HAMD-17: 8–17	/	MA (9); fluoxetine (9)	Male: (35.5 ± 5.4); female: (33 ± 4)	LR3	MA, 30 min, once per day, fluoxetine 20 mg/d; for 1 month.	GE Sigma 3.0T; RS
Yi (44)	Depression (42)	DSM-IV	RCT	7.8 ± 1.4 m; 8.0 ± 1.3 m; 7.6 ± 1.1 m	HAMD-17: 19.6 ± 2.51; 18.9 ± 3.01; 18.4 ± 2.61	HAMD-17: 12.5 ± 3.44; 12.2 ± 2.75; 8.0 ± 2.83	Fluoxetine (14); MA (14); fluoxetine+MA (14)	(33.6 ± 8.4); (35.5 ± 7.4); (37.0 ± 8.6)	LR3	MA, 30 min, once per day, fluoxetine 20 mg/d; for 1 month.	GE Sigma 3.0T; RS
Yi et al. (45)	Depression (26); HC (13)	DSM-IV	Pre–post	More than 2 weeks	HAMD-17: 18–24	/	MA (13); SA (13)	18–60	LR3	MA, needing three times, 0–1, 6–7, and 12–13 min.	GE Sigma 3.0T; RS
Quah-Smith et al. (46)	MDD (10); HC (10)	DSM-IV	Pre–post	/	Mean score, BDI: 22.8; MADRS: 21; HAMD-17; 18.5	/	MDD (10); HC (10)	43.7/39.8	LR14, LR8, CV14, and HT7	LA, 4J laser energy.	3T; RS
Li (47)	MDD (16)	CCMD-3	RCT	17.62 ± 16.83; 15.87 ± 10.48	HAMD-24: 28.37 ± 5.47; 29.75 ± 4.59	HAMD-24: 9.75 ± 4.77; 16.12 ± 3.64	MA+paroxetine (8); SA+paroxetine (8)	(40.12 ± 13.04); (34.12 ± 11.72)	GV20, DU24, LI4, and LR3	MA, 30 min, 3 times/w; paroxetine hydrochloride 20 mg/d; for 12 weeks.	Siemens 3 T; RS
Deng et al. (48)	MDD (16)	DSM-IV	Crossover design	/	HAMD-17: >18	/	EA; SA	NA	GV20	EA, 20 min.	Siemens 3.0T/NRER
Huang (49)	Depression (9)	ICD-10	Observational studies	/	HAMD-24: 34.19 ± 9.94; 32.65 ± 6.67	HAMD-24: 7 ± 4.75; 14 ± 8	EA (9)	NA	GV20 and EX-HN3	EA, 3 times/week for 8 weeks.	Siemens 3.0T; RS
Qu (50)	Mild-to-moderate primary depression (12); HC (11)	ICD-10	Pre–post	/	HAMD-17: 18.9 3 ± 1.93	/	EA (12); HC (11)	(25.0 ± 9.28); (21.5 ± 1.78)	GV20 and EX-HN3	EA, for 30 min.	GE Sigma 3.0T; RS
Wang et al. (51)	Depression (15); HC (15)	ICD-10	Pre–post		HAMD-17: 17–35		MA (15); HC (15)	18–65	ST36, SP6, LR3, PC6, HT7, and GV20	MA, 5 times/w, a total of 40 times.	GE Sigma 3.0T; RS
Ye (52)	Depression (36)	CCMD-3; DSM-IV	RCT	/	SDS: 60 ± 2.34; 59.58 ± 2.73; MADRS: 22.95 ± 1.74; 22.83 ± 2.16	SDS: 36.63 ± 2.08; 46.32 ± 1.69; MADRS: 5.44 ± 1.27; 14.06 ± 1.03;	MA+fluoxetine (18); SA+fluoxetine (18)	(44.50 ± 2.46); (43.78 ± 2.146)	RN12, RN10, RN6, RN4, ST24, and ST26	MA, 30 min, once every 2 days, for 3 months, 2 days off every 4 weeks.	Siemens 3.0T; RS
Deng et al. (53)	MDD (29); HC (29)	DSM-IV	Pre–post	/	HDRS-17: 21.31 ± 2.58; SDS: 62.72 ± 9.81; SAS: 62.14 ± 8.79	/	EA (29)	(28.69 ± 6.69); (26.76 ± 1.72)	GV20	EA, 20 min at GV20.	Siemens 3.0T/NRER
Yang et al. (54)	MDD (80)	DSM-IV	RCT	14.10 ± 16.48; 16.70 ± 19.07	HAMD-24: 28.75 ± 4.13; 28.80 ± 4.04; SDS: 68.08 ± 5.70; 68.22 ± 4.50	HAMD-24: 17.38 ± 5.64; 20.03 ± 5.22; SDS: 56.10 ± 6.30; 60.08 ± 5.25	EA (40); sham EA (40)	(29.83 ± 8.62); (30.85 ± 8.07)	GV20, EX-HN3, LI4, PC6, SP6, and HT7	Fluoxetine, 20 mg/d, once per day, for 14 days; EA 30 min, once daily for 14 days.	Philips, 3.0T; RS
Wang et al. (55)	Depression (46)	ICD-10	RCT	/	MADRS: 22.94 ± 7.36; 22.83 ± 9.17; SDS: 47.83 ± 6.46; 47.44 ± 9.23	MADRS: 5.44 ± 5.37; 14.06 ± 4.39; SDS: 26.83 ± 6.46; 34.94 ± 5.40	MA+fluoxetine (22); SA+fluoxetine (24)	(44.5 ± 10.47); (43.78 ± 9.10)	RN12, RN10, RN6, RN4, KL17, ST24, and extra-point	Fluoxetine, 20 mg/d, once per day plus MA or SA; once a day for the first 3 days and subsequently once every 3 days for 8 weeks.	Siemens 1.5 T; RS
Li et al. (56)	Depression (198)	ICD-10	RCT	11.6 ± 1. 9; 11. 2 ± 1.7	HAMD-17: 19.99 ± 4.02; 19.81 ± 3.97	HAMD-17:15.32 ± 0.88; 17.87 ± 0.95	EA+paroxetine (99); paroxetine (99)	(34.6 ± 2.6); (34.9 ± 2.3)	GV20, EX-HN3, DU16, GB20, DU14, PC6, and SP6	EA, 30 min, 3 times/w; paroxetine 10 mg/d, adjusted to 20 mg/d; for 6 weeks.	NA; RS
Li (57)	MDD (7)	ICD-10	RCT	/	HAMD-24: 23.71 ± 4.07	HAMD-24: 5.14 ± 3.24	EA+placebo drug (2); EA+escitalopram (3); SA+escitalopram (3)	27.57	GV20, EX-HN3, LR3, PC6, SP6, HT7, and ST36	EA, 30 min, 3 times/w; escitalopram; for 8 weeks.	Siemens 3.0T; RS
Wang et al. (58)	MDD (46)	ICD-10	RCT	/	MADRS: 22.94 ± 7.36; 22.83 ± 9.17; SDS: 47.83 ± 6.46; 47.44 ± 9.23	MADRS: 5.44 ± 5.37; 14.06 ± 4.39; SDS: 26.83 ± 6.46; 34.94 ± 5.40	MA+fluoxetine (22); SA+fluoxetine (24)	44.5; 43.78	RN12, RN10, RN6, RN4, KL17, ST24, and extra-point	MA, once a day; fluoxetine, 20 mg/d; for 8 weeks.	Siemens 1.5 T; RS
Yang (59)	Geriatric depression (26); HC (20)	ICD-10	RCT	2–12 m	HAMD-17: 22.07 ± 2.71; 21.38 ± 3.12 SDS: 67.07 ± 8.84; 64.46 ± 7.434	HAMD-17: 12.27 ± 2.99; 12.08 ± 2.84 SDS: 42.60 ± 5.73; 45.54 ± 5.25	MA (13); fluoxetine (13)	(55.40 ± 5.70); (53.31 ± 4.46); (42.33 ± 8.19)	GV20, EX-HN1, EX-HN3, EX-HN5, GB20, LI4, LR3, PC6, SP6, and ST36	MA, 30 min, 5 times/w; fluoxetine 20 mg/d, 5 times/w; for 8 weeks.	GE Sigma 3.0T; RS
Duan et al. (60)	MDD (30); HC (29)	DSM-V	Pre–post	/	HAMD-17: 21.31 ± 2.58; SDS: 62.72 ± 9.81	/	EA (30)	(28.69 ± 6.69); (26.76 ± 1.72)	GV20	EA, 20 min	Siemens 3.0 T; NRER
Wang et al. (61)	Depression (60); HC (30)	DSM-V	RCT	4.2 ± 0.8 y; 4.4 ± 0.9 y	HAMD-17: 20.8 ± 2.9; 21.1 ± 3.1; BDI: 13.5 ± 1.7; 13.3 ± 1.8	HAMD-17: 11.3 ± 1.6; 12.9 ± 1.7; BDI: 7.2 ± 0.8; 8.3 ± 1.0	MA+venlafaxine (30); venlafaxine (30)	32 ± 8	GV20 and EX-HN3	MA, 30 min, once every other day; venlafaxine 75 mg/d at week 1, with increasing by 225 mg/d; for 12 weeks.	Siemens 3.0 T; RS

*CCMD diagnostic criteria: at least four of the following symptoms were included: (1) loss of interest and no sense of pleasure; (2) loss of energy or fatigue; (3) psychomotor hysteresis or agitation; (4) low self-evaluation, self-blame, or sense of guilt; (5) difficulty in association or decreased ability of conscious thinking; (6) repeated thoughts of death or suicidal or self-injurious behavior; (7) sleep disorders, such as insomnia, early awakening, or excessive sleep; (8) decreased appetite or weight loss; and (9) loss of libido*.

*CCMD, Chinese Classification of Mental Disorders; BDI, Beck Depression Inventory; EA, electroacupuncture; GV20, RS, resting state; Baihui; EX-HN3, Yintang; d, day; min, minutes; HAMD, Hamilton Depression Scale; w, week; ICD, International Classification of Diseases; HC, healthy controls; MA, manual acupuncture; RCT, randomized controlled trial; RN12, Zhongwan; RN10, Xiawan; RN6, Qihai; RN4, Guanyuan; extra-point, Qipang; LA, laser acupuncture; DSM, Diagnostic and Statistical Manual of Mental Disorders; LR3, Taichong; m, months; NRER, non-repeated event-related; LI4, Hegu; LR8, Ququan; CV14, Juque; HT7, Shenmen; SA, sham acupuncture; DU24, Shenting; ST36, Zusanli; SP6, Sanyinjiao; PC6, Neiguan; RWS, real-world study; SDS, Self-Rating Depression Scale; ST24, Huaroumen; ST26, Waiguan; KL17, Shangqu; GB20, Fengci, DU16, Fengfu; DU14, Dazhui, and EX-HN1, Sishecong*.

Study sample sizes ranged from 29 to 160, with a total of 826 depression patients and 416 health controls (HCs). For diagnostic criteria of depression, DSM-IV or DSM-V criteria were used in nine studies (44, 45, 48, 52–54, 60, 61), ICD-10 was used in nine studies (41, 49–51, 55–58), and CCMD-3 was used in four studies (40, 42, 47, 52).

In terms of depression types, MDD was diagnosed in nine studies (41, 46–48, 53, 54, 57, 58, 60), geriatric depression was diagnosed in one study (59), mild-to-moderate primary depression was diagnosed in one study (44), mild depression was diagnosed in one study (43), and the remaining were not specified.

In this study, non-repeated event-related (NRER) paradigm was adopted in three studies (42, 53, 60), and a resting-state fMRI (rs-fMRI) paradigm was used in the remaining studies.

The main treatment procedures included manual acupuncture (MA), EA, and LA, with sessions ranging from 4 to 12 weeks. The top three acupoints were GV20-Baihui (13), EX-HN3-Yintang (10), and LR3-Taichong (7). Twenty-two studies reported the needle retention time of their main interventions, with ~12 min for average duration.

### Results of MRI Studies

Regional homogeneity (ReHo), (fractional) amplitude of low-frequency fluctuation [(f)ALFF], and FC were applied in 22 studies; whereas magnetic resonance spectroscopy (MRS), sMRI, and diffusion tensor imaging (DTI) were only used in two studies (Tables 2–**5**).

**Table 2:** The fMRI studies of acupuncture on depression.

**Number**	**References**	**Seed regions**	**Groups**	**Treatments (*n*)**	**Results**
**ReHo**					
1	Wang (42)	Whole brain	Depression (19); HC (19)	MA (19)	(i) **MA (effective):** left superior frontal gyrus, left middle frontal gyrus, left inferior frontal gyrus, left posterior cerebellar lobe, right posterior cerebellar lobe, left anterior central gyrus, left lenticular nucleus, and left inferior parietal lobule √. (ii)**MA (non-effective):** left posterior cerebellar lobe, right posterior cerebellar lobe, and right superior temporal gyrus √.
2	Deng et al. (48)	Whole brain	MDD (16)	EA (NA); SA (NA)	**EA vs. SA:** left ventromedial prefrontal lobe, left insula, left anterior cingulate gyrus, right thalamus, right superior temporal gyrus, bilateral precuneus, bilateral anterior cerebellar lobe, and right posterior cerebellar lobe ↑.
3	Qu (50)	Whole brain	Depression (12); HC (11)	EA (12)	**EA:** right middle frontal gyrus, supramarginal and angular gyrus, and left middle temporal gyrus ↑; right caudate nucleus ↓.
4	Yang et al. (54)	Whole brain	MDD (80)	EA (40); SA (40)	**EA vs. SA:** right inferior occipital gyrus, inferior temporal gyrus, middle temporal gyrus, orbital gyrus, dorsolateral and medial superior frontal gyrus, left postcentral gyrus, supramarginal gyrus, anterior and posterior cingulate gyrus, supramarginal gyrus, occipital lobe, right cerebellum, and limbic system ↑; left hippocampus, left parahippocampal gyrus, left amygdala, left thalamus, left lenticular nucleus, right caudate nucleus, and bilateral angular gyrus↓.
**fALFF/ALFF**					
1	Yi et al. (43)	Whole brain	Depression (18)	MA (9); Fluoxetine (9)	**MA:** left frontal lobe, right frontal lobe, left occipital lobe, right middle occipital lobe, left precuneus, and posterior cingulate gyrus ↓.
2	Yi (44)*	Whole brain	Depression (42)	Fluoxetine (14); MA (14); fluoxetine+MA (14)	(i)**MA+fluoxetine:** left frontal lobe, right frontal lobe, bilateral inferior parietal lobule, precuneus, posterior cingulate gyrus, left occipital lobe, and right middle occipital lobe ↓. (ii) **MA:** left frontal lobe, right frontal lobe, left occipital lobe, right occipital lobe, left precuneus, and posterior cingulate ↓. (iii) **Correlation**: positive correlation between the left frontal lobe, left middle frontal gyrus, left frontal lobe, left parietal lobe and left occipital lobe, and the final HAMD score.
3	Yi et al. (45)	Whole brain	Depression (26); HC (13)	MA (13); SA (13)	(i) **MA:** right frontal lobe of control group, and in right superior frontal gyrus, right middle frontal gyrus, left superior frontal gyrus, bilateral inferior parietal lobule, right precuneus, and left anterior cingulate cortex ↓. (ii) **SA:** bilateral inferior parietal lobule and left occipital lobe. ↓.
4	Li (47)	Whole brain	MDD (16)	MA+paroxetine (8); SA+paroxetine (8)	(i) **MA+paroxetine:** left orbitofrontal cortex, bilateral anterior cingulate gyrus, left caudate nucleus, bilateral hippocampal, parahippocampal gyrus, and right medial prefrontal cortex ↑. bilateral insula and thalamus ↓. (ii) **SA+paroxetine:** right anterior cingulate gyrus, and left orbitofrontal cortex ↑, the right insula ↓. (iii)**MA+paroxetine vs. SA+paroxetine:** bilateral orbitofrontal cortex, bilateral anterior cingulate gyrus, left caudate nucleus, right hippocampus, and left parahippocampal gyrus ↑; left cuneus, left dorsolateral prefrontal cortex, right temporal pole, right insula, right middle temporal gyrus, and right cerebellum ↓.
5	Qu (50)*	Whole brain	Depression (12); HC (11)	EA (12); HC (11)	**EA**: right precuneus and middle frontal gyrus ↑.
6	Wang et al. (51)	Whole brain	MDD (15); HC (15)	MA (15); HC (15)	**MA vs. HC:** inferior temporal gyrus, fusiform gyrus, parahippocampal gyrus, uncinate, and limbic lobe ↑; bilateral parietal lobe and precuneus lobe ↓.
7	Yang et al. (54)	Whole brain	MDD	EA (40); SA9 (40)	(i) **EA:** left orbitofrontal lobe, bilateral anterior cingulate gyrus, left caudate nucleus, bilateral hippocampal/parahippocampal gyrus and right medial prefrontal cortex↑; bilateral insula, and bilateral thalamus ↓. (ii) **EA vs. SA:** bilateral orbitofrontal lobe, bilateral anterior cingulate gyrus, left caudate nucleus, right hippocampus, and left parahippocampal gyrus ↑; left cuneus, left dorsolateral prefrontal cortex, right temporal pole, right insula, right middle temporal gyrus, and right cerebellum ↓.
8	Li et al. (56)	Whole brain	Depression (198)	EA+paroxetine (99); paroxetine (99)	(i) **EA+paroxetine:** left orbitofrontal lobe, anterior cingulate gyrus, left caudate nucleus, and hippocampus ↑; bilateral thalamus and insula ↓. (ii) **Paroxetine:** left orbitofrontal and anterior cingulate gyrus ↑; right insula ↓.
9	Li (57)	Whole brain	MDD (7)	EA+placebo drug (2); EA+escitalopram (3); escitalopram+SA (3)	(i) **EA+placebo drug:** bilateral pre-central gyrus, cingulate gyrus and superior frontal gyrus, left postcentral gyrus, middle frontal gyrus, insula and superior temporal gyrus, right medial frontal gyrus, and inferior frontal gyrus √. (ii) **Escitalopram+SA:** bilateral pre-central gyrus, postcentral gyrus, superior frontal gyrus and medial frontal gyrus, left anterior cingulate gyrus and middle temporal gyrus, right middle frontal gyrus, caudate nucleus, and posterior cerebellar lobe √. (iii) **EA+escitalopram:** bilateral anterior cerebellum, left insula, right parietal lobule, cingulate gyrus, thalamus, and inferior frontal gyrus √.
10	Yang (59)*	Whole brain	Depression (15); HC (15)	MA (15)	Right middle frontal gyrus, left hippocampus, right putamen, left thalamus, and right amygdala ↑; right thalamus ↓.
**FC**					
1	Yi et al. (45)	Left ACC	Depression (26); HC (13)	MA (13); SA (13)	**MA:** bilateral parietal lobe, right temporal lobe, left posterior cingulate gyrus, right superior frontal gyrus, left middle frontal gyrus, and caudate nucleus ↑.
2	Huang (49)	DLPFC	Depression (9)	EA (9)	**EA:** left superior temporal gyrus ↓. **Correlation:** between brain cognitive control network and seven factor subdivision rate of HAMD-24 scale.
3	Ye (52)	pa, ph, vmh	Depression (36)	MA+ Fluoxetine (18); SA+ Fluoxetine (18)	(i) **MA:** For pa: rsFC between right middle occipital gyrus, left middle occipital gyrus, left anterior central gyrus, and left paracentral lobule ↓. For ph: right superior cerebellum and the left superior cerebellum ↓. For wmh: rsFC between left middle gyrus, left module lobe, left anterior central gyrus, left h-corner inferior frontal gyrus, and right posterior central gyrus ↓. (ii) **SA:** For left pa and ph: -. For left vmh: rsFC between right fusiform gyrus, the right orbital superior frontal gyrus, the right glume middle gyrus, the left angular gyrus, and the right angular gyrus ↓. (iii) **MA vs. SA**: For left vmh: -.
4	Deng et al. (53)	PC/PCC	MDD (29); HC (29)	EA (29)	Bilateral ACC ↑; left middle prefrontal cortex, left angular gyrus, and bilateral HIPP/paraHIPP ↓.
5	Wang et al. (55)	Amygdala	Depression	MA+fluoxetine (22); SA+fluoxetine (24)	(i) **MA+fluoxetine:** For left amygdala: subgenual anterior cingulate cortex (sgACC)/pregenual anterior cingulate cortex (pgACC) ↑; For right amygdala: paraphippocampus (Para)/putman (Pu) ↑. (ii) **SA+fluoxetine:** For right amygdala: left Para/Pu ↓. (iii) **MA+fluoxetine vs. SA+fluoxetine:** For left amygdala: left sgACC/pgACC ↑; For right amygdala: left Para/Pu ↑. (iv) **Correlation**: negative correlation between rsFC in the amygdala-sgACC/pgACC with MADRS and SDS.
6	Wang et al. (58)	Ventral and dorsal striatal regions	MDD (46)	MA+fluoxetine (22); SA+fluoxetine (24)	**MA+fluoxetine vs. SA+fluoxetine:** corticostriatal reward circuits ↑; the striatal-cerebellar regions ↓. **Correlation:** negative correlation rsFC in the inferior ventral striatum and medial prefrontal cortex, ventral rostral putamen and amygdala/parahippocampus with MADRS and SDS.
7	Duan et al. (60)	amygdala	MDD (30); HC (29)	EA (30)	**EA:** **For left amygdala:** the bilateral PG, PAG, left anterior insula, right posterior insula, right precuneus, and right dACC ↓; the right OFC and left dorsolateral prefrontal cortex (DLPFC) ↑.
**ICA**					
1	Quah-Smith et al. (46)	ICA (NA)	MDD (10); HC (10)	LA (10)	**LA:** wider posterior DMN modulation at the parieto–temporal–limbic cortices.

*ReHo, regional homogeneity; vs., versus; ↑, increase; ↓, decrease. √, change; fALFF, fractional amplitude of low-frequency fluctuation; ACC, anterior cingulate cortex; DLPFC, dorsolateral prefrontal cortex; pa, paraventricular nucleus of hypothalamus; ph, posterior hypothalamus; vmh, ventromedial hypothalamic nucleus; PC/PCC, precuneus/posterior cingulate cortex; OFC, orbital frontal cortex; HIPP, hippocampi; MADRS, Montgomery–Åsberg Depression Rating Scale; SDS, Self-Rating Depression Scale; PG, precentral gyrus; AG, angular gyrus; PAG, periaqueductal gray; ICA, independent component analysis*.

* *Studies that were included in the meta-analysis*.

These studies included five different comparisons: (1) acupuncture vs. sham acupuncture (SA); (2) acupuncture plus drug vs. SA plus drug; (3) acupuncture vs. drug; (4) acupuncture plus drug vs. drug; and (5) post-acupuncture vs. pre-acupuncture.

The main findings were as follows:

(i) ReHo was used in four studies (42, 48, 50, 54), showing that acupuncture could modulate ReHo value in limbic system and cerebral cortex (Table 2).
(ii) ALFF was used in 10 studies (43–45, 47, 50, 51, 54, 56, 57, 59): these studies showed relatively consistent results that acupuncture mainly modulates brain activity in the cerebellum, limbic lobe, frontal lobe, temporal lobe, and thalamus (Table 2).
(iii) Seed-based FC was used in seven studies (45, 49, 52–54, 58, 60), whereas independent component analysis (ICA) was used in one study (46) (Table 2). Specifically, Wang et al. (55) showed that acupuncture enhanced FC between the amygdala and anterior cingulate cortex (ACC); Deng et al. (53) showed that acupuncture enhanced FC between the precuneus/posterior cingulate cortex (PC/PCC) and ACC; and one study (45) selected the ACC as the seed and found that acupuncture enhanced FC between the ACC and the bilateral parietal lobe, right temporal lobe, left posterior cingulate gyrus, etc. Therefore, acupuncture could increase FC between the ACC and other brain regions.
(iv) Proton MRS (^1^H-MRS) was used in two studies (40, 41), which showed increased *N*-acetylaspartate/creatine (NAA/Cr) ratio after EA treatment (Table 3). DTI was used in two studies (59, 61) (Table 4), and voxel-based morphometry (VBM) was used in two studies (57, 59) (**Table 5**), but none of these studies was able to detect significant neurological changes after acupuncture.
(v) In addition, a total of seven (45, 47, 48, 52, 54, 55, 58) were conducted to compare the brain response of verum acupuncture (VA) with SA on depression. The relatively consistent findings argue that acupuncture can modulate more brain activity, especially in the brain regions associated with depression compared with SA. In addition, two studies comparing acupuncture plus drugs with SA plus drugs found that the combination of acupuncture with drugs has a complex mutual influence on the central nervous system, rather than simply a combination of curative effects.

**Table 3:** ^1^H-MRS studies of acupuncture on depression.

**Number**	**References**	**Seed regions**	**Groups**	**Treatments (*n*)**	**Results**
1	Duan (40)	Left and right hippocampus and frontal lobe	Depression (75)	Fluoxetine (25); EA (25); fluoxetine+EA (25)	(i)**NAA/Cr** **EA:** bilateral hippocampus ↑. **Fluoxetine+EA:** bilateral hippocampus ↑. (ii) **Cho/Cr EA:** bilateral frontal lobe↓. **Fluoxetine+EA:** bilateral frontal lobe ↓.
2	Duan et al. (41)	Hippocampus	MDD	Fluoxetine+EA (34); fluoxetine (36);	**Fluoxetine+EA:** hippocampal volume -; NAA/Cr ↑. **Correlation:** positive relevance between the hippocampal Cho/Cr change ratio, hippocampal NAA/Cr change ratio, and reduction rate of the HAMD score.

*↑, increase after treatment; ↓, decrease after treatment; -, no statistical difference before and after treatment; ^1^H-MRS, proton magnetic resonance spectroscopy; HAMD, Hamilton Depression Scale; EA, electroacupuncture*.

**Table 4:** DTI studies of acupuncture on depression.

**Number**	**References**	**Analytical methods**	**Groups (n)**	**Treatments (n)**	**Results**
1	Yang (59)	DTI (FA, MD)	Depression (26); HC (20)	MA (13); fluoxetine (13)	**MA:** FA: -; MD: -.
2	Wang (61)	DTI (FA)	Depression (60); HC (30)	MA+venlafaxine (30); venlafaxine (30)	**MA+venlafaxine vs. venlafaxine** FA: bilateral frontal lobes, bilateral inferior temporal gyrus, and bilateral deep temporal occipital region ↑.

*DTI, diffusion tensor imaging; FA, fractional anisotropy; MD, mean diffusivity; EA, electroacupuncture; HC, health controls; MA, manual acupuncture*.

**Table 5:** The sMRI studies of acupuncture on depression.

**Number**	**References**	**Analytical method**	**Groups**	**Treatments (*n*)**	**Results**
1	Li (57)	VBM	MDD (7)	EA+placebo drug (2); EA+escitalopram (3); escitalopram+sham EA (3)	**EA+placebo drug:** gray matter volume: central anterior gyrus, middle temporal gyrus, and lower temporal gyrus √.
2	Yang (59)	VBM	Depression (26); HC (20)	MA (13); fluoxetine (13)	**MA:** -.

*sMRI, structural magnetic resonance imaging; VBM, voxel-based morphometry; MDD, major depressive disorder; EA, electroacupuncture*.

### Results of Meta-Analysis

**Figure 2:**
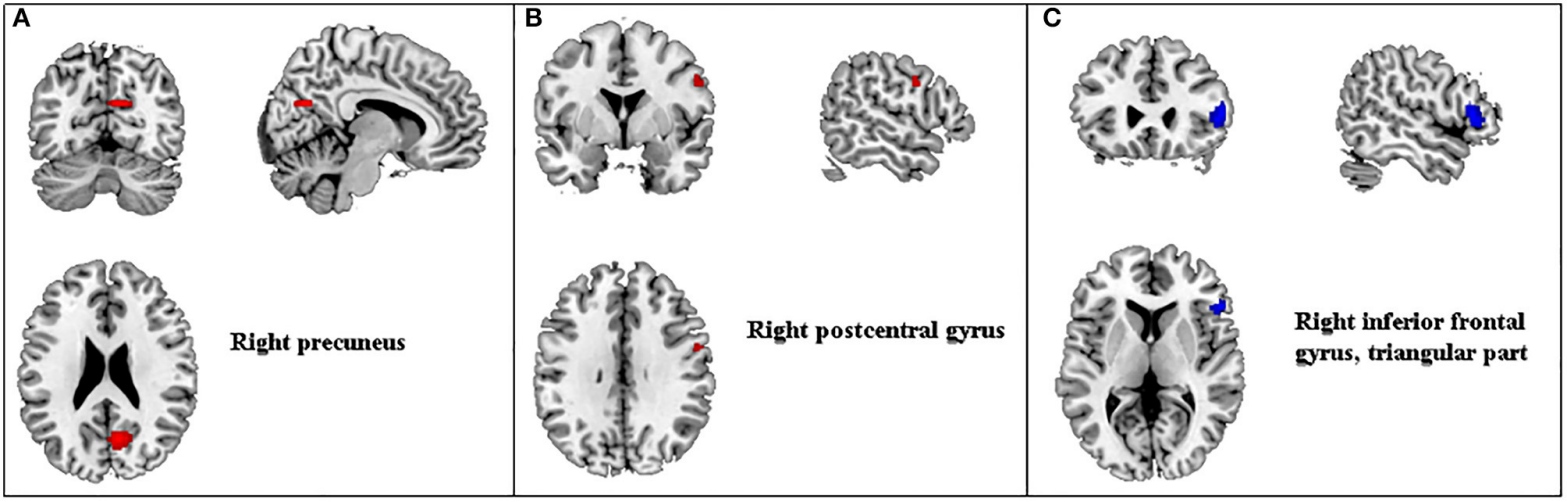
Regions of ALFF differences before and after acupuncture treatment in patients with depression. Red for increased ALFF **(A,B)** and blue for decreased ALFF **(C)**. ALFF, amplitude of low-frequency fluctuation.

Three studies (44, 50, 59) met the criteria for meta-analysis. A total of 86 depressed patients were compared before and after acupuncture treatment for ALFF changes. This analysis revealed ALFF signals in the right precuneus and right postcentral gyrus significantly increased, and ALFF signals in the right inferior frontal gyrus (IFG) decreased in depressed patients after acupuncture treatment (Table 6 and Figures 2A–C).

**Table 6:** Clusters of ALFF differences before and after acupuncture on depression.

**Brain regions**	**MNI coordinate**	**SDM-Z value**	***p*-value**	**Number of voxels**
Right precuneus, BA 23	8, −66, 24	2.718	<0.01	122
Right postcentral gyrus, BA 6	56, 0, 32	2.203	<0.05	36
Right inferior frontal gyrus, triangular part, BA 45	52, 26, 6	−2.743	<0.01	168

*MNI, Montreal Neurological Institute; SDM, signed differential mapping; BA, Brodmann's area; ALFF, amplitude of low-frequency fluctuation*.

## Discussion

This systematic review was designed to summarize findings of MRI studies aimed at evaluating the neurological effects of acupuncture treatment for depression. A total of 22 studies involving 810 depression patients and 416 HCs were evaluated. Importantly, further meta-analysis using SDM-PSI was conducted with three studies to explore changes in ALFF after acupuncture for depression.

### Characteristics of MRI Study on Acupuncture Therapy for Depression

Among the 22 studies included in this study, 21 studies were from China, which may be explained by the fact that acupuncture originated in China over 3,000 years ago and is widely accepted in the Chinese society.

It is a common phenomenon in MRI research for the sample size of a study to be small. Although the optimal sample size needed to detect or evaluate an experimental factor has been investigated in this field (62, 63), the number of subjects is usually limited by practical constraints such as scanning time and costs (64). To reduce subject bias, it is necessary to estimate size effects, between- and within-subject variances, and temporal autocorrelation matrix (65).

In regard to the classification of depression, the patients in eight studies were considered MDD, one study examined geriatric depression, and the rest of the patients were not specified. Indeed, depression is a heterogeneous syndrome that includes a wide variety of symptoms and different responses to treatment (66). At present, several studies have shown that depression subtypes are highly correlated with brain function, which may help identify individuals who would benefit most from a particular treatment (66, 67). Our systematic review was unable to find a study that investigates the relationship between subtypes of depression and efficacy of acupuncture based on MRI techniques. Thus, future studies are needed to do this.

Regarding the types of acupuncture, MA, EA, and LA are those most often used as treatment for depression. They are different acupuncture techniques. Specifically, MA is defined as manual manipulation of needles after insertion at certain acupuncture points (16). EA is characterized by application of small current passing through acupuncture needles to produce the combined effect of electrical and needling stimulation (68). LA is a kind of non-penetrating acupuncture that uses low-power laser to stimulate acupoints (16). Anyway, all of them can stimulate acupoints. Although some studies (69–73) have compared the clinical efficacy of MA, EA, and LA for diseases, little research has been conducted to compare the differences in brain activity induced by each of the three types of acupuncture on patients using MRI methods.

The first two most common acupoints are GV20 and EX-HN3, which are located in Du meridian. Du meridian is responsible for regulating consciousness disease and organ lesions (74). GV20 is located on the highest place of the head where all the Yang meridians meet. Based on TCM theory, acupuncture on GV20 is used to clear the mind, lift the spirit, and tonify Yang (75). EX-HN3 is in charge of nourishing the brain and regulating emotional disorder (76). Moreover, animal studies have shown that the release of depression symptoms with acupuncture on GV20 and EX-HN3 may be related to decreasing serum corticosterone concentrations and increasing neurotransmitter levels (5-HT, Glu, and GABA) and protein levels of brain-derived neurotrophic factor (BDNF) (77, 78). In addition, three studies only applied one acupoint for treatment, whereas the rest combined multiple acupoints for treatment of depression. Recent studies (79, 80) have shown that a combination of acupoints can activate more areas of the brain as compared with a single acupoint. In the future, it will be important to investigate the relationship among GV20 and EX-HN3, efficacy, and activation of brain regions.

In addition, it is very hard to distinguish the activation effect of specific acupoints on the brain since combinations of different acupoints will cause some confusion, although we really want to perform them. Therefore, we summarized as much as possible studies on treating depression with the same single acupoint. Firstly, three studies (43–45) investigated brain response of acupuncture at LR3 and revealed that acupuncture could reduce ALFF in the frontal lobe, precuneus, and occipital lobe. Secondly, two studies (53, 60) involved FC of acupuncture at GV20 and demonstrated that acupuncture could modulate abnormal default mode network (DMN) in patients and could affect the FC of the amygdala. Thirdly, three studies (48, 52, 54) were conducted to investigate brain activity of acupuncture at GV20 and EX-HN3 and showed that acupuncture not only remodeled the white matter fiber bundle microstructure in certain brain regions but also increase NAA/Cr ratio, regulated FC of cognitive control networks, and activated brain activity of the middle temporal gyrus and caudate nucleus.

### Experimental Designs of MRI

The fundamental experimental design of fMRI research mainly includes task-state fMRI and rs-fMRI. The earliest studies of acupuncture using fMRI were performed with a block design to observe the immediate effects of acupuncture (81–83). The needle inserted before the scan was stimulated continuously for several blocks of 30 s to 2 min. However, based on TCM theory, acupuncture induces a lasting effect that will still produce the corresponding neurological response even after holding for 30 min (82, 84). Thus, in recent years, some studies have adopted NRER designs (85–87); these designs are more in line with acupuncture methods and their effects. They also can reduce interference from the persistent effect of acupuncture that occurs when a single, prolonged acupuncture stimulation is given during the scanning process (88). However, this type of experimental design may have the limitation of a single stimulation, which is different from clinical treatment.

In a word, more and more attention is being paid to the effects of acupuncture on rs-fMRI (89, 90). This procedure of resting state is relatively simple and has several advantages that are more suitable for exploring mechanism of acupuncture effect. For example, brain function characteristics or changes in different states, such as before, during, and after acupuncture treatment, and especially any long-term cumulative effects, can be obtained through different data processing methods. Moreover, in recent years, rs-fMRI has been increasingly used to explore the effective neural mechanism of acupuncture for some diseases, such as Alzheimer's disease (91), stroke (92), and migraine (93). Rs-fMRI study is considered to be more advantageous than task fMRI in neuropsychiatric diseases (94). The above also explains why the number of rs-fMRI in this study is significantly higher than that of task states.

### Study Design

Well-designed research trials are critical for determining the efficacy and effectiveness of new interventions (95). In interventional study designs, which are a subset of experimental study designs, researchers apply treatment interventions or preventive services to patients and then examine outcomes (96). The randomized controlled trial (RCT) design is typically considered as the “gold standard” for ascertaining intervention efficacy and effectiveness (97). There are other interventional study designs, including pre–post study design, non-randomized trial study design, and crossover RCT study design (80, 81).

In this review study, the experimental study design of acupuncture therapy for depression included RCT and pre–post study designs. RCTs with a placebo arm control have high internal validity and are considered a reliable method of evaluating treatment efficacy (98, 99). Only seven studies adapted placebo arms as controls. In interventional trials, blindness is often necessary, especially for patient-reported outcomes, to prevent reporting bias. However, blinding is difficult to implement due to the special nature of acupuncture, which can reduce the credibility of the research results. This is especially true for studies in which the effectiveness of acupuncture or acupuncture combined with drugs is being compared with drug treatment alone. Although this pre–post design has the disadvantages of enhanced selection bias, detection bias, and performance bias, it may be valuable to explore the first steps in the efficacy of new therapies (phase I design) at a time of increased demand for services and reduced resources (100). Thus, if the RCT is designed well, the only difference between study groups is the intervention itself (101). However, this type of design is pretty difficult to be conducted in an acupuncture study. Thus, finding a more rigorous design is an important guarantee for reaching reliable conclusions.

### Analytical Methods of MRI

The most commonly used method is fMRI with ALFF and FC. ALFF is associated with blood oxygen level-dependent (BOLD) signals and can be used to detect a spontaneous, intrinsic neuronal activity (102), which has been applied in bipolar disorder patients (103), obsessive-compulsive disorder (104), and MDD (105). In our meta-analysis, we found that acupuncture could increase ALFF signals in the right precuneus and right postcentral gyrus and decreased ALFF signals in the right IFG. One meta-analysis (106) displayed increased ALFF in the bilateral precuneus of MDD patients compared with HCs. Moreover, the precuneus is a key node in the DMN (107), and low right precuneus activity has been associated with more depressive episodes in MDD patients, indicating a deleterious effect of depressive episodes on DMN (107). Several studies found that the ALFF values in the right inferior frontal significantly increased in depressive patients compared with HCs (106, 108). What is more, the IFG, as a key region in the emotion–cognition interplay (109), is involved in processing emotional information and evaluating affective salience (110). Therefore, increased ALFF signals in the right precuneus and increased ALFF signals in the IFG might be the underlying mechanism for the effects of acupuncture in depressed patients. Two previous studies (111, 112) found the fALFF in the left postcentral gyrus was significantly reduced in MDD patients. However, in our study, increased ALFF values in the right postcentral gyrus were identified. In view of these differences, additional research is necessary to confirm whether acupuncture treatment for depression produces consistent changes of ALFF values.

FC refers to the temporal correlation between spatially remote neurophysiological events (113). In this study, to understand the seed-based resting-state FC regarding acupuncture therapy for depression, the seed points selected were the left ACC, dorsolateral prefrontal cortex, posterior hypothalamus (ph), ventromedial hypothalamic nucleus (vmh), paraventricular nucleus of hypothalamus (pa), PC/PCC, amygdala, and striatum. Due to differences in the selection of seed points, the functional connections detected in various studies were also inconsistent, with the exception of the ACC, which was identified with a relatively high degree of consistency. Interestingly, a recent study (114) supported the notion of the ACC as a promising predictor of antidepressant response, which further illustrates the importance of the ACC in the pathogenesis of depression and as a target of acupuncture treatment for depression. The ACC, a limbic structure, is associated with a range of other limbic and related regions, including the amygdala and orbitofrontal cortex (OFC), involved in emotional and reward-related processing (107, 115). Collectively, these findings may give us some clues whether the effects of acupuncture on emotion-related diseases are related to its effect on the ACC.

The fMRI analytical method was the most widely used in the studies evaluated in this review, while sMRI analytical method was used relatively infrequently. One reason is that in recent years, more and more attention has been paid to fMRI and less to sMRI. The other reason is that acupuncture is more likely to modulate brain functional activity in a short period of time, whereas structural alternations may not be easy to form or be detected. However, different modalities of neuroimaging can provide information complementary to each other (116). Therefore, the important implication of this study is that maybe we should explore more analytical methods to better explain the underlying neural mechanisms of acupuncture treatment for depression.

### Comparing Verum Acupuncture With Sham Acupuncture on the Brain Response

In this study, we found that VA could activate more brain activity and increase connectivity than SA. Although the superiority of VA over SA remains a global controversy, an increasing number of neuroimaging studies suggest that compared with SA, VA works in a more targeted and specialized manner on depressive patients (47, 52, 54), which is consistent with other diseases, such as migraine (117).

In clinical trials, placebo controls should be consistent with active treatment (that is, reaching the equivalence of blindness), except that they are physiologically inert (118). In regard to acupuncture, however, it is difficult to develop placebo needles satisfying both blinding and physiological inertness. At present, the devices of SA can be divided into two types based on whether they penetrate the skin. However, due to a less effective form of penetrating SA, it is rarely used anymore. Several non-penetrating devices including a foam placebo device (119), Streitberger placebo device (120), and Park device (121), have been developed to evaluate potential placebo effects. Nevertheless, not only are the effects of SA caused by psychological effects of the sham procedure, but also data from imaging studies have shown that expectations, learning, and background factors play important roles in the placebo effect (122–124).

Indeed, placebo research has revealed that the definition of placebo may not be as clear as necessary for clinical trials in non-pharmacological fields. The placebo effect is seen as a positive and useful factor in treatment, especially in clinical practice, which is considered a part of every routine treatment (125). Thus, it is unnecessary to argue about how much treatment effect depends on placebos. Instead, the most important question should focus on clinical efficacy: what will relieve patients' pain and how best to treat them. This pragmatic approach aims to maximize all the positive aspects of treatment while minimizing the risks and negative effects, including the nocebo effect (125).

### Limitations

Although this review provides a detailed overview of the current literature about MRI research on the neurological effects of acupuncture therapy for depression, some limitations need to be noted. Firstly, most of the included studies are from Chinese literature, which reduces the readability of research literature and the applicability to other races. Secondly, the brain responses to acupuncture are influenced by many factors, for example, acupuncture depth, duration and course, differences in MRI devices, analytical methods, experimental designs, and patients' responses to acupuncture. Therefore, it is difficult to determine the specific factor that affects the brain during acupuncture treatment for depression. Researchers should follow the standards for reporting interventions in controlled trials of acupuncture (STRICTA) guidelines (126) when designing and reporting MRI of acupuncture research. Thirdly, due to the small samples and large heterogeneity among different acupuncture types, acupoints, design, and analytical methods, it is extremely hard to perform a comprehensive meta-analysis for more studies to draw consistent conclusions. It is essential that researchers report relevant coordinates for further deep analysis and investigation. In addition, it is extremely hard to explore how FC was altered by acupuncture treatment for depression since different seed points were involved in related studies. Fourthly, only three studies explored the correlation between brain responses to acupuncture and clinical outcomes. Anyway, these correlations play an important role in understanding biomarkers on acupuncture treatment for depression. Finally, most of the studies did not set SA as a placebo control, making it impossible to avoid placebo effects. In addition, the sample size was also small, which limited the reliability of MRI mechanism research on the mechanism of acupuncture treatment of depression.

## Conclusions

In conclusion, MRI analytical methods of acupuncture treatment for depression include ^1^H-MRS, ReHo, (f)ALFF, FC, VBM, and DTI. Among them, fMRI was the often most used and showed that acupuncture could modulate brain function in several ways, while sMRI and DTI were used the least and did not detect any significant changes. Moreover, the relatively consistent fMRI results showed increased NAA/Cr ratio, increased ALFF in the right precuneus, decreased ALFF in the IFG, and increased FC of the ACC. However, future studies need to apply more analytical methods of MRI to investigate the neurological effects of acupuncture treatment in depression in the future. In addition, improved report specifications, well-designed experiments, consistent analytical methods, and larger sample sizes will enable the field to better elucidate the underlying mechanisms of acupuncture therapy for depressed patients.

## Author Contributions

JZ and JX designed the whole study, analyzed the data, and wrote the manuscript. XW and DN searched and selected the studies. JL and YZ participated in the interpretation of data. HY and QH offered good suggestions. All authors read and approved the final manuscript.

## Conflict of Interest

The authors declare that the research was conducted in the absence of any commercial or financial relationships that could be construed as a potential conflict of interest.

## Publisher's Note

All claims expressed in this article are solely those of the authors and do not necessarily represent those of their affiliated organizations, or those of the publisher, the editors and the reviewers. Any product that may be evaluated in this article, or claim that may be made by its manufacturer, is not guaranteed or endorsed by the publisher.
