# Supplementary material for: Magnetic Resonance Imaging Studies on Acupuncture Therapy in Depression: A Systematic Review
Source: Front Psychiatry. 2021 Aug 20;12:670739. doi: 10.3389/fpsyt.2021.670739 (PMC8417590; doi:10.3389/fpsyt.2021.670739)
Supplement: Supplementary file 1 [file Table_1.DOCX]

| **Supplementary Table 1. search strategy for PubMed and CNKI.** | | | |
| --- | --- | --- | --- |
| **Databases** | **Coverage** | **Searches** | **Results** |
| PubMed Database | Date of inception  – October 20, 2020 | 1. ((((((((((((((((((((((MRI[Title/Abstract]) OR (rs-fMRI[Title/Abstract])) OR (fMRI[Title/Abstract])) OR (functional connectivity[Title/Abstract])) OR (task fMRI[Title/Abstract])) OR (ReHo[Title/Abstract])) OR (ALFF[Title/Abstract])) OR (VBM[Title/Abstract])) OR (DTI[Title/Abstract])) OR ((structural magnetic resonance imaging[Title/Abstract]) OR (sMRI[Title/Abstract])) OR MRI[Title/Abstract])) OR (MRS[Title/Abstract])) OR (magnetic resonance spectroscopy[Title/Abstract])) OR (blood oxygen level dependent[Title/Abstract])) OR (surface based morphometry[Title/Abstract])) OR (cortical volume[Title/Abstract])) OR (gray matter volume[Title/Abstract])) OR   (ractional anisotraphy[Title/Abstract])) OR (mean diffusivity[Title/Abstract])) OR (white matter[Title/Abstract])) OR (surface area[Title/Abstract]))   1. (((depression[Title/Abstract]) OR (depressive disorder[Title/Abstract])) OR (major depression disorder[Title/Abstract]))) 2. (((acupuncture therapy[Title/Abstract]) OR (acupuncture[Title/Abstract])) OR (electroacupuncture[Title/Abstract])) OR (moxibustion[Title/Abstract])   4. #1 AND #2 AND #3 | 34 |
| Chinese Nation Knowledge Infrastructure  (CNKI, Chinese Database) | Date of inception  – October 20, 2020 | [(主题=针刺+灸法+针灸+针+电针+针法) AND (主题=磁共振+MRI+功能磁共振+弥弥散张量成像+体素+功能连接+局部不一致+结构磁共振+磁共振波 +DTI+BOLD+Reho+MRS) AND (主题=抑郁+重度抑郁障碍+抑郁障碍)](https://er.szlib.org.cn/KNS8/AdvSearch?id=40&dbcode=SCDB&searchtype=gradeSearch&ishistory=1" \o "(主题=针刺 + 灸法 + 针灸 + 针 + 电针) AND (主题=磁共振 + MRI + fMRI + DTI + VBM + 功能连接 + ReHo) AND (主题=抑郁 + 重度抑郁障碍)" \t "https://er.szlib.org.cn/rwt/331/https/NNYHGLUDN3WXTLUPMW4A/KNS8/manage/_blank) | 102 |
